# Longitudinal Analysis of Peripheral MicroRNA Expression and Depressive Symptom Severity Change in a Community Cohort

**DOI:** 10.3390/epigenomes10020035

**Published:** 2026-06-02

**Authors:** Jan Dahrendorff, Chengqi Wang, Agaz Wani, Zachary Graham, Allison E. Aiello, Annie Qu, Derek E. Wildman, Monica Uddin

**Affiliations:** 1 Department of Global, Environmental, and Genomic Health Sciences, College of Public Health, University of South Florida, Tampa, FL 33612, USA; dahrendorff@usf.edu (J.D.); chengqi@usf.edu (C.W.); dwildman@usf.edu (D.E.W.); 2 USF Genomics Program Sequencing Core, College of Public Health, University of South Florida, Tampa, FL 33612, USA; 3 Robert N. Butler Columbia Aging Center, Department of Epidemiology, Mailman School of Public Health, Columbia University, New York, NY 10032, USA; 4 Department of Statistics and Applied Probability, University of California Santa Barbara, Santa Barbara, CA 93106, USA

**Keywords:** microRNA expression, depressive symptom severity, genome-scale analysis, community-based cohort

## Abstract

Background: Depression is a heterogeneous and recurrent condition, whose underlying biological mechanisms remain poorly understood. MicroRNAs (miRNAs), small non-coding RNAs that regulate post-transcriptional gene expression, are increasingly implicated in cross sectional miRNA studies of depression and depressive symptoms; however, longitudinal studies capturing miRNA changes over time in relation to depression are scarce. Methods: We conducted small RNA sequencing of leukocyte-derived miRNAs at two timepoints in a prospective community-based cohort (*n* = 185) to assess associations between within-person changes in depressive symptom severity (ΔPHQ-9) and longitudinal miRNA expression. Differential expression analyses were performed using a paired limma-voom framework, adjusting for covariates (baseline PHQ-9, sex, age, DNAm-derived immune cell covariates and ancestry components derived from matched blood samples) and within-subject correlation. Results: Although no miRNAs survived multiple-testing correction, 68 mature unique miRNAs showed nominal associations (*p* < 0.05) with depressive symptom severity change (ΔPHQ-9). Several top candidates, including miR-493-3p, miR-409-3p, and miR-323a-3p, displayed expression patterns aligning with prior reports implicating these miRNAs in stress responsivity, synaptic plasticity, and neurodevelopmental regulation. Exploratory follow-up of predicted targets of the nominally symptom-associated miRNAs converged on genes in pathways central to depression biology, including neurotransmission, HPA axis/inflammatory signaling, neuroplasticity, and circadian regulation. Enrichment analyses highlighted receptor tyrosine kinase and intracellular signaling cascades, hypothalamic–pituitary–adrenal axis feedback, and inflammatory pathways. Conclusions: These findings provide preliminary evidence that peripheral miRNA expression changes may reflect depressive symptom trajectories, highlighting potential molecular pathways involved in depression. Further studies with larger samples and broader symptom severity are warranted to validate these dynamic miRNA signatures.

## 1. Introduction

MicroRNAs (miRNAs) are short, ~22 nucleotide non-coding RNA molecules that serve as important post-transcriptional regulators of gene expression [1]. These small non-coding RNAs dynamically regulate gene expression by binding to partially complementary sequences on target messenger RNAs, resulting in mRNA degradation or inhibition of translation [2]. Although not coding for peptides or proteins, miRNAs modulate >60% of protein-coding genes in humans making these molecules an important regulator of gene function [3]. Briefly, RNA polymerase II transforms an intragenic or intergenic miRNA gene into a pre-miRNA [4]. Subsequently, through a series of cleavage and maturation events, the pre-miRNA is transported to the cytoplasm where it integrates with RNA-induced silencing complexes (RISCs). These miRNA sequences guide the RISC to target mRNA transcripts, inducing translational cleavage or repression of mRNA [4]. Due to their imperfect sequence recognition, miRNAs can bind to multiple genes and regulate their expression [4]. The contribution of aberrant miRNAs to disease pathology has been documented in a wide range of diseases including cancers [5], immune disorders [6], metabolic diseases [7], cardiovascular disease [8], neurological [9], and neurodegenerative [10] diseases as well as psychiatric disorders [11,12].

miRNAs have been increasingly evaluated in relation to the pathophysiology of major depressive disorder (MDD) with a large body of research implicating the involvement of miRNAs in the dysregulation of several pathways associated with MDD (summarized in [11]). For example, miRNAs have been implicated in the dysregulation of monoamines [13], alterations in neuroplasticity [14], changes in hypothalamic–pituitary–adrenal axis function [15], and aberrant inflammatory responses [16,17]. Additionally, miRNAs have been associated with relapse status in patients with a history of recurrent MDD [18], explored as a potential therapeutic target [19,20], and evaluated for their utility as biomarkers of treatment outcomes [21].

The majority of studies to date remain cross-sectional comparisons of miRNA profiles between patients with MDD and healthy controls [22], providing important initial insights into potential “depression-associated” miRNAs. However, existing studies are limited as they offer only a static snapshot of a dynamic disorder, which preclude conclusions regarding whether differential miRNA profiles observed in depression represent antecedent risk markers or downstream consequences of the disorder. For example, in a recent investigation of 62 adolescents with depression, small RNA sequencing of dried blood spots identified nine circulating miRNAs that differentiated depressed youths from controls; notably, baseline levels of three miRNAs (miR-3613-5p, miR-30c-2, miR-942-5p) predicted greater depression severity at follow-up assessments years later [23]. Similarly, Belzeaux and colleagues applied small RNA sequencing in 237 depressed patients and identified two previously unrecognized miRNAs (miR-3688 and miR-5695) that predicted worsening suicidal ideation during an eight-week course of duloxetine or placebo [24]. While this work highlights the potential of miRNA profiling to forecast symptom trajectories, they do not address whether miRNAs themselves dynamically change during treatment.

Only a select few studies to date have profiled miRNAs at multiple timepoints, often evaluating miRNA changes in response to an intervention such as antidepressants or psychotherapy [17,18,25]. For example, Lopez et al. applied small RNA sequencing in a placebo-controlled antidepressant trial of 258 patients with MDD and found that four miRNAs (miR-146a-5p, miR-146b-5p, miR-24-3p, miR-425-3p) changed significantly from baseline to eight weeks in patients who responded to treatment, but not in non-responders [26]. Similarly, Yrondi et al. (2020) employed next-generation small RNA sequencing to longitudinally profile miRNA expression in 160 patients with MDD undergoing treatment with escitalopram [27]. Blood samples were collected at baseline and again after two weeks of treatment, enabling a within-subject analysis of dynamic changes in circulating miRNAs. The authors identified 356 miRNAs, of which 45 showed significant differential expression following treatment. This study provides evidence that antidepressant exposure can induce alterations in peripheral miRNA expression, highlighting both the potential role of miRNAs in mediating drug response and also the need for more studies employing longitudinal sampling [27].

Taken together, these findings underscore how unbiased sequencing approaches can reveal novel associations with symptom change, emphasizing the need for longitudinal, genome-scale study designs. To our knowledge, no prior study has directly assessed the relation between within-person changes in depressive symptom severity with genome-scale alterations in miRNA expression in a community-based setting [22]. To address this gap, we investigated whether changes in depressive symptom severity within community-dwelling individuals were associated with corresponding changes in peripheral miRNA expression levels over time, using genome-scale analytic approaches.

## 2. Results

### 2.1. Study Participants

Descriptive statistics for demographic characteristics and depressive symptom severity are shown in Table 1. To identify miRNAs whose expression trajectories were associated with changes in depression severity, we conducted small RNA sequencing to profile leukocyte-derived miRNA expression in 306 samples from 185 study participants, focusing on participants with complete depressive symptom severity data.

### 2.2. Differential Expression of miRNA and Depressive Symptom Severity

After quality control, filtering of low-abundance features, and normalization, 542 mature miRNAs were analyzed using limma-voom, adjusting for covariates including baseline PHQ-9 symptom severity.

No miRNA reached the FDR threshold (*q* < 0.05, minimum observed *q* = 0.0802). However, 76 (68 unique mature) miRNAs showed nominal associations (*p* < 0.05) with within-person change in depressive symptom severity (ΔPHQ-9). Given that 542 miRNAs were tested, approximately 5% of features would be expected to reach *p* < 0.05 by chance alone (~27 miRNAs), and thus these nominal associations should be interpreted cautiously. Of these, 37 miRNAs (32 unique mature) had positive ΔPHQ-9 coefficient (β_3_) values, indicating higher expression among participants whose symptoms worsened (higher ΔPHQ-9), while 39 miRNAs (36 unique mature) had negative ΔPHQ-9 coefficient (β_3_) values, indicating higher expression among participants whose symptoms improved (lower ΔPHQ-9). miRNAs with the biggest effect sizes included hsa-miR-363-3p (AveExpr = 7.42; ΔPHQ-9 coefficient (β_3_) = +0.029; *p* = 5.1 × 10^−4^; *q* = 0.0802; higher in worsening), hsa-miR-493-3p (AveExpr = 2.03; ΔPHQ-9 coefficient (β_3_) = −0.056; *p* = 5.4 × 10^−4^; *q* = 0.0802; higher in improvement), hsa-miR-411-5p (AveExpr = 3.62; ΔPHQ-9 coefficient (β_3_) = −0.048; *p* = 7.0 × 10^−4^; *q* = 0.0802; higher in improvement), hsa-miR-127-3p (AveExpr = 3.68; ΔPHQ-9 coefficient (β_3_)= −0.050; *p* = 8.0 × 10^−4^; *q* = 0.0802; higher in improvement), and hsa-miR-409-3p (AveExpr = 4.89; ΔPHQ-9 coefficient (β_3_) = −0.046; *p* = 8.0 × 10^−4^; *q* = 0.0802; higher in improvement). Additional miRNAs with similarly strong associations, including hsa-miR-323a-3p, hsa-miR-432-5p, and hsa-miR-337-5p, are presented in Table 2.

These results indicate that while some miRNAs (e.g., hsa-miR-363-3p) increase with worsening symptoms, several of the most robust candidates show higher expression among participants with symptom improvement. Top 10-ranked candidates are shown in Table 2. The full list of the nominally significant miRNAs in the differential expression analysis results is provided in Supplementary Table S1. Additional tables showing the top 10 differentially expressed miRNAs associated with symptom worsening and symptom improvement are provided in Supplementary Tables S2 and S3.

### 2.3. Exploratory Target Prediction of Differentially Expressed miRNA

To gain insight into the potential functional consequences of these miRNA changes, we examined predicted target genes using in silico analysis of the 68 unique, mature nominally associated miRNAs (*p* < 0.05) with the mirDIP database. At the Very High confidence threshold, this yielded 5573 unique predicted target genes after collapsing at the miRNA–gene level. In sum, 2782 genes were targeted by ≥2 unique miRNAs while 556 genes were targeted by ≥5. Notably, several RNA-binding and neurodevelopmental regulators emerged among the most convergently targeted genes, including *NFIB* (23 miRNAs), *QKI* (20 miRNAs), and *MBNL1* (19 miRNAs), consistent with roles in neuronal splicing and plasticity. Table S4 shows the top 10 genes with the most convergently targeted by nominally associated miRNAs.

**Figure 1:**
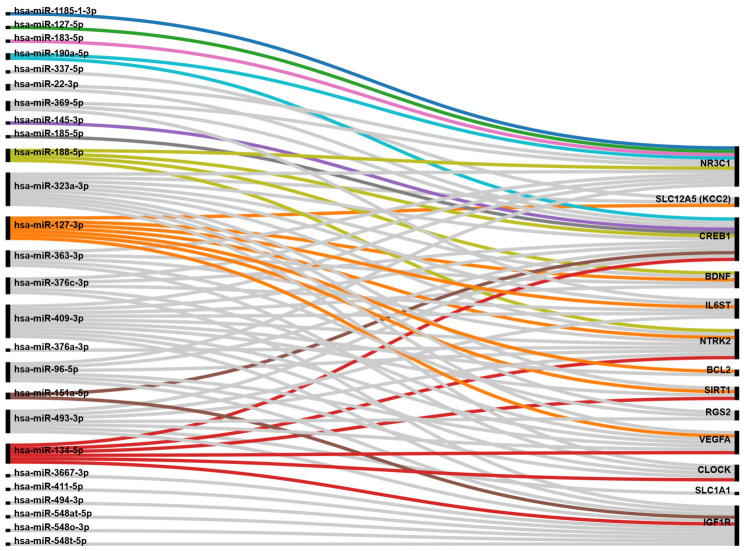
Convergent targeting depression-relevant genes by nominally associated miRNAs. Sankey diagram depicting select mature miRNAs (**left**) nominally associated with within-person changes in depressive symptom severity and their predicted target genes (**right**) with prior links to depression biology (e.g., *NR3C1*, *SLC12A5/KCC2*, *CREB1*, *BDNF*, *IL6ST*, *NTRK2*, *BCL2*, *SIRT1*, *RGS2*, *VEGFA*, *CLOCK*, *SLC1A1*, *IGF1R*). The Edges (links) represent unique miRNA–gene prediction pairs retrieved from *mirDIP* at the Very High confidence threshold (top 1%), deduplicated at the mature miRNA–gene level to remove precursor redundancy. Node bar and link widths reflect the number of connections (degree); genes targeted by more distinct miRNAs appear thicker. Colored flows highlight selected miRNAs, whereas grey flows denote additional miRNAs within the panel. This visualization illustrates convergent regulation, where multiple miRNAs target shared transcripts (e.g., *CREB1* and *NR3C1*), consistent with coordinated post-transcriptional control of pathways central to MDD such as neurotransmission, HPA axis and inflammatory signaling, neuroplasticity, circadian regulation, and trophic or vascular support.

To contextualize predicted targets, we examined the complete list of genes targeted by our 68 unique, nominally significant, mature miRNAs (Supplementary Table S5) and highlighted the genes most consistently implicated in pathways known to be important in depression (Figure 1) drawing on evidence from prior comprehensive reviews [34,35,36]. Convergent targeting was observed across multiple functional domains including neuronal transporters and receptors (*SLC12A5/KCC2*, 3 miRNAs; *SLC1A1*, 1 miRNA), neuroplasticity-related genes (*BDNF*, 5; *NTRK2*, 9; CREB1, 13), stress and immune regulators (*NR3C1*, 12; *IL6ST*, 6), metabolic and trophic factors (*SIRT1*, 4; *IGF1R*, 12), and circadian/survival genes (*CLOCK*, 5; *BCL2*, 2). Additional inflammatory and neurotrophic mediators such as *VEGFA* (7) and *RGS2* (3) were also repeatedly targeted (Figure 1). Taken together, these convergent hubs and functionally relevant targets map onto pathways central to depression biology, including neurotransmission, HPA axis/inflammatory signaling, neuroplasticity, and circadian regulation [34].

### 2.4. Gene Ontology Analysis

Our enrichment analysis of GO biological processes enriched among genes targeted by the implicated miRNAs highlighted developmental and morphogenetic programs, with top terms including embryonic forelimb morphogenesis (FDR = 1.36 × 10^−3^; fold enrichment = 1.60), forelimb morphogenesis (FDR = 1.86 × 10^−3^; 1.54), and lung epithelium development (FDR = 4.32 × 10^−3^; 1.54). Additional signals reflected regulation of cellular dynamics, such as positive regulation of epithelial cell migration (FDR = 1.89 × 10^−3^; 1.51), regulation of epithelial cell migration (FDR = 4.56 × 10^−4^; 1.47), and stem cell proliferation (FDR = 4.09 × 10^−3^; 1.41). Consistent with post-transcriptional control by miRNAs, we also observed enrichment for regulation of mRNA stability (FDR = 1.68 × 10^−4^; 1.33) and related RNA metabolic terms. Collectively, these results implicate pathways involved in tissue morphogenesis, growth-factor responses, cell motility, and RNA stability (Top 15 shown in Figure S1). Full results of all over-representation tests and pathways analyses are in Supplementary Tables S6–S10.

The investigation of enrichment among molecular functions pointed to post-transcriptional and transcriptional regulation with mRNA 3′-UTR binding (FDR = 3.43 × 10^−4^; 1.40) and mRNA binding (FDR = 1.85 × 10^−6^; 1.29) among the leading signals, alongside chromatin- and TF-related activities such as chromatin binding (FDR = 7.70 × 10^−5^; 1.19), DNA-binding transcription activator activity, RNA polymerase II-specific (FDR = 3.74 × 10^−4^; 1.21), and DNA-binding transcription repressor activity (FDR = 2.08 × 10^−2^; 1.20). We also noted adaptor/readout functions (e.g., signaling adaptor activity) consistent with receptor-proximal signaling (Top 15 shown in Figure S2).

The results for our analysis of cellular components revealed strong enrichment for ribonucleoprotein and synaptic structures, including cytoplasmic ribonucleoprotein granule (FDR = 6.03 × 10^−3^; 1.22), P-body (FDR = 8.31 × 10^−3^; 1.33), and nuclear speck (FDR = 2.16 × 10^−3^; 1.19). Synaptic compartments were among the most robust signals: postsynapse (FDR = 5.53 × 10^−8^; 1.21), postsynaptic specialization (FDR = 7.71 × 10^−5^; 1.23), postsynaptic density (FDR = 9.95 × 10^−4^; 1.22), and asymmetric synapse (FDR = 1.05 × 10^−4^; 1.23) (Top 15 shown in Figure S3).

*PANTHER* pathway analysis identified two pathways that reached FDR < 0.05: EGF receptor signaling (fold enrichment = 1.29; FDR = 0.0318) and gonadotropin-releasing hormone receptor signaling (fold enrichment = 1.20; FDR = 0.0227). These represent the only statistically significant PANTHER pathways detected in our dataset.

**Figure 2:**
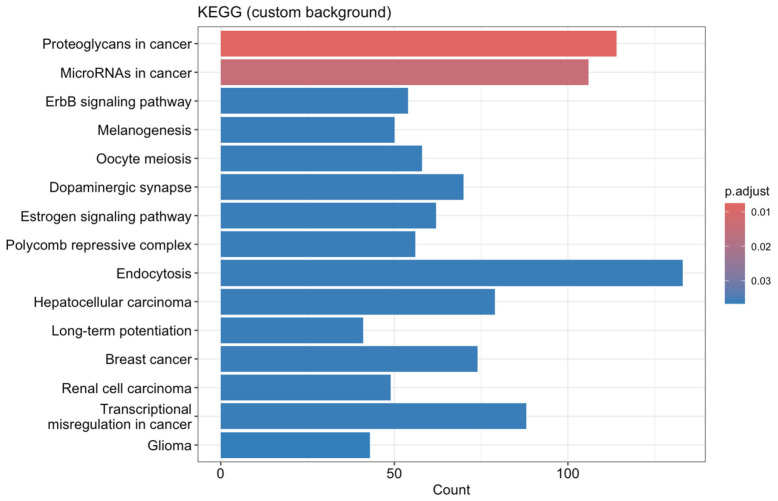
KEGG pathway enrichment of predicted gene targets of miRNAs whose expression was significantly associated with within-person changes in depressive symptom severity. The analysis compared the foreground set (targets of 68 symptom-associated miRNAs) against a custom background of predicted targets of all 542 mature miRNAs expressed in the dataset. The bar plot displays the top 15 enriched pathways ranked by significance, with bar length representing log_2_ (Fold Enrichment) and bar color indicating statistical significance (−log_10_ FDR).

Lastly, *KEGG* analysis identified numerous overrepresented pathways among miRNA-targeted loci, including receptor-mediated signaling, neurotransmission, and transcriptional regulation, with statistically significant (FDR < 0.05) categories including ErbB signaling, estrogen signaling, dopaminergic/glutamatergic synapse, endocytosis, proteoglycans in cancer, transcriptional misregulation in cancer, and miRNAs in cancer. Figure 2 presents the top 15 statistically significant KEGG pathways. Given that enrichment analyses were based on predicted targets of nominally associated miRNAs, these results should be interpreted cautiously and are intended to identify potential biological pathways for future validation rather than to provide confirmatory evidence of pathway involvement.

## 3. Discussion

In this exploratory longitudinal analysis of community-dwelling adults, we conducted small RNA sequencing to characterize blood-derived miRNA expression changes associated with changes in depressive symptom severity (ΔPHQ-9) over time. To our knowledge, this is among the first studies to leverage a paired, within-subject design in a community sample to link genome-scale miRNA expression changes with symptom change. Prior human miRNA studies of depression have largely been cross-sectional case-control studies profiling candidate miRNA expression [22]. Our study design leveraging paired baseline and follow-up samples allowed us to examine whether intra-individual changes in depression symptom severity were associated with corresponding longitudinal shifts in circulating miRNA levels.

### 3.1. Differential Expression of miRNA and Depressive Symptom Severity

We identified 76 (68 unique mature) nominally significant miRNA expression changes associated with changes in depressive symptom severity. Among our top-ranked were several miRNAs previously implicated in the regulation of stress or neurodevelopmental processes [22]—pathways that play important roles in depression and depressive symptom development [34,37]. For example, MiR-493-3p, a top candidate associated with symptom improvement, has been linked to depression-related phenotypes in preclinical models [29]. Zhou and colleagues reported that miR-493-3p was significantly upregulated in the hippocampus of rats exposed to chronic unpredictable stress. In our study, greater symptom improvement was associated with higher expression in circulating miR-493-3p, and therefore its upregulation may potentially reflect symptom resolution in humans.

Additionally, miR-409-3p showed a negative ΔPHQ-9 coefficient (β_3_) in our data, indicating higher expression among participants whose symptoms improved. This pattern aligns with prior work reporting miR-409-3p downregulation in depressive states [32]. The downregulation of hsa-miR-409-3p has previously been associated with adolescent depression in peripheral blood [32] and in brain samples of suicide completers [38]. Other studies have implicated miR-409-3p in cortical neurodevelopment regulating genes central to neuronal integrity and synaptic plasticity [39,40], pathways repeatedly documented to be dysregulated in MDD [41,42,43]. Dysregulation of such neurodevelopmental programs could potentially contribute to mood disorder vulnerability. In our analysis, miR-409-3p exhibited a negative ΔPHQ-9 coefficient (β_3_) (−0.046), indicating higher expression among individuals whose symptoms improved, consistent with its reported involvement in neuronal integrity and synaptic plasticity [44]. These convergent lines of evidence may suggest miR-409-3p merits further study as a potential dynamic marker of brain-related plasticity in depression.

A third notable hit nominally associated with the change in depressive symptom severity in our study was hsa-miR-323a-3p, which was upregulated among participants whose symptoms improved. This miRNA has been linked to MDD in human brain tissue [33]. Fiori and colleagues identified hsa-miR-323a-3p to be upregulated in the anterior cingulate cortex and habenula in post-mortem samples of individuals with MDD and showed it directly suppresses the *ErbB4* (neuregulin) receptor in neuronal cells [33]. The *ErbB4-neuregulin* pathway is crucial for synaptic development, maintenance, and plasticity, processes that are central to mood regulation and psychiatric disorders [45]. The authors further complemented their analysis of post-mortem samples with mechanistic experiments in mice and reported that overexpression of miR-323a-3p in the anterior cingulate cortex increased anxiety- and depressive-like behaviors, whereas its inhibition exerted the opposite effect [33]. Another study using a chronic mild stress mouse model reported the upregulation of miR-323a-3p in the hippocampus to be associated with depression-like behaviors [46]. Experimentally induced inhibition of miR-323a-3p (via knockdown) significantly ameliorated depression-like behaviors, restored hippocampal monoamine levels, and attenuated neuronal apoptosis in a chronic stress model [46], illustrating how miRNA modulation could yield mechanistic insights into potential candidate therapeutic targets for MDD. These findings highlight miR-323a-3p as a regulator of neuronal signaling pathways relevant to depression and underscore its potential as a mechanistic link between altered miRNA expression and depression phenotypes.

Taken together, the nominal associations we observed for miR-493-3p, miR-409-3p, and miR-323a-3p—miRNAs that appear among the top-ranked differentially expressed miRNAs in relation to depressive symptom change in our study—align with prior evidence linking these candidates to stress responsivity, synaptic plasticity, and neuronal survival, processes repeatedly implicated in the pathophysiology MDD [47]. While none of the signals withstood multiple-testing correction, the partial overlap with independent human and rodent studies suggests that these miRNAs may warrant further investigation as potential contributors to depressive symptom trajectories, pending replication in larger samples with more broader ranges in depressive symptom severity.

### 3.2. Predicted Targets of Differentially Expressed miRNA and Pathway Enrichment

To explore the potential functional consequences of the observed miRNA changes, we examined predicted gene targets of the symptom-associated miRNAs and their functional enrichments. Importantly, these analyses rely on in silico target prediction and enrichment of nominally associated miRNAs and are therefore exploratory and intended to generate hypotheses regarding potential biological mechanisms. Our initial analyses of predicted targets revealed notable convergence on RNA-binding proteins, splicing regulators, and transcription factors especially *NFIB*, *QKI*, and *MBNL1*, which were targeted by the greatest number of symptom-change-associated miRNAs. The complex interactions of miRNAs and these regulatory genes could amplify subtle transcriptional changes into broader downstream effects on many interconnected pathways of depression [48,49].

The analysis of predicted gene targets also revealed convergence on several canonical pathways often implicated in MDD. In particular, several predicted targets included genes central to neurotransmission (e.g., *SLC12A5*, *SLC1A1*), neurotrophic signaling (*BDNF*, *NTRK2*, *CREB1*), and stress and immune regulators (*NR3C1*, *IL6ST*)—all pathways extensively documented to play a critical role in the pathophysiology of depression [34,50]. Among the predicted targets, several genes critical for neurotransmission and maintenance of excitatory/inhibitory balance were identified including *SLC12A5* and *SLC1A1*. Converging evidence indicates that dysregulation of these transporters contributes to impaired synaptic plasticity, excitatory/inhibitory imbalance, and stress-related dysregulation in MDD [34,51,52]. Consistent with these target-level findings, our pathway enrichment analyses further highlighted receptor tyrosine kinase and intracellular signaling cascades (e.g., *PI3K–Akt* and *MAPK/ERK*), suggesting that these miRNA–gene interactions converge on molecular pathways regulating synaptic function and stress responsivity [53].

Within this framework, *BDNF* and its receptor *NTRK2* act as upstream regulators that signal through the PI3K-Akt and *MAPK/ERK* and have been linked to modulate key MDD pathways such as neuronal survival, synaptic plasticity, and neurogenesis [54]. Reduced *BDNF* levels are consistently observed in patients with MDD [55] and in animal models of depression [56] while *BDNF-TrkB* signaling has been documented to play an important role for antidepressant efficacy [57]. Evidence from candidate gene miRNA studies documents a complex regulation of *BDNF* and *CREB1* by several miRNAs [22,58] highlighting the significance of this pathway in relation to depressive symptom severity.

Similarly, several stress- and immune-regulatory targets including *NR3C1* and *IL6ST* emerged among our target-related findings. Both play important roles in HPA axis feedback and cytokine signaling, processes repeatedly implicated in MDD pathophysiology [15,50]. Dysregulation of these pathways can reduce glucocorticoid receptor sensitivity, removing feedback inhibition on the HPA axis and facilitating neuroinflammatory cascades that impair synaptic and neurotrophic signaling [59,60]. Together, these findings align with enrichment results highlighting HPA axis and receptor-mediated signaling pathways, suggesting coordinated miRNA regulation of stress–immune processes in depression. Relatedly, additional enriched targets, including *SIRT1*, *IRS1*, and *IGF1R*, underscore the interface between metabolic and neuroplastic mechanisms, while circadian and apoptotic regulators such as *CLOCK*, *MAPK14*, and *BCL2* point to rhythm-related and stress-response pathways involved in neuronal vulnerability [61,62].

The findings from our pathway enrichment further underscore the potential role of these miRNA targets in depression biology, highlighting that individual gene signals might coalesce into broader neuroplasticity and stress-response pathways. For example, predicted miRNA targets were significantly enriched for neurodevelopmental and synaptic plasticity pathways serving critical roles in the pathophysiology of depression mood [63]. Increasing evidence documents the role of miRNAs in the regulation of such pathways [14,43] emphasizing how miRNAs’ dysregulation may shape depression pathogenesis [14] and even treatment response [43]. Taken together, these findings suggest that even modest miRNA changes may influence important pathways of neuroplasticity, neurotransmitter balance, stress hormone sensitivity, and immune signaling that may underlay changes in depressive symptom severity.

### 3.3. Limitations

This study has several limitations that warrant consideration. First, our community-based sample reported relatively low depression severity at both assessments (Table 1). A low symptom burden potentially reflects a more subtle biological signal, meaning any changes in circulating miRNA expression associated with depressive symptom change might have been accordingly nuanced. The restricted range of PHQ-9 scores may have constrained the detectable within-subject miRNA differences. To overcome this challenge, we made deliberate analytic decisions to filter low-abundance miRNAs to mitigate noise. Nevertheless, studies in depression research often use a PHQ-9 score of 10 or higher as a clinical threshold for identifying MDD in research [64,65]. Although we focused on depressive symptom severity rather than MDD, individuals with higher depression symptom severity—which are not well represented in our cohort—might have more pronounced alterations in specific circulating miRNAs than what we observed here. For example, individuals without a depressive disorder typically show PHQ-9 scores around 3, whereas those with MDD average scores in the mid-to-high teens. Thus, our largely asymptomatic cohort may exhibit only modest shifts in miRNA levels with changes in mood, potentially underestimating or obscuring the stronger miRNA expression differences that might be observed in populations with more severe depression. Second, the two-year interval between our two measurement timepoints introduces uncertainty about the temporal stability and persistence of the observed miRNA expression changes [66]. It remains unclear whether peripheral miRNA signals related to depression represent enduring trait markers or rather reflect transient state-dependent shifts. Prior research on circulating miRNAs suggests that many miRNAs can remain relatively stable within individuals over short periods (weeks to months) but also that certain miRNAs can fluctuate substantially even over a span of a few months [66]; however, the durability of miRNA expression changes over multi-year intervals is largely unexamined. Our two-year gap between assessments means that any within-subject associations between miRNA levels and depression severity could be influenced by unmeasured intervening factors or interim fluctuations. Therefore, the detected miRNA changes might reflect ephemeral state-related variations present at the time of each assessment, rather than stable, long-lasting alterations. Future studies incorporating more frequent sampling or additional timepoints could clarify whether peripheral miRNA biomarkers of depression are truly durable over years or primarily capture short-term state changes in mood and physiology. Lastly, this study leveraged a population-based, community-representative cohort from Detroit, Michigan, designed to reflect the sociodemographic composition of the population. As such, race, geographic context, and socioeconomic characteristics are intrinsic features of the study design rather than sources of bias. The findings are therefore most directly generalizable to similar urban, community-based populations. Replication in other cohorts will be important for evaluating the broader applicability of these results.

## 4. Materials and Methods

### 4.1. Study Participants and Blood Sampling

This study leveraged data from a subset of study participants from the Detroit Neighborhood Health Study (DNHS), a prospective population-based longitudinal cohort of predominantly African American adults (≥18 years) in Detroit, Michigan [67,68]. The DNHS was established to investigate how biological variation, neighborhood factors, and psychosocial stressors shape risk for psychopathology. Participants were recruited in 2008–2009 and followed annually until 2013. Structured telephone interviews captured information on neighborhood perceptions, mental and physical health status, social support, trauma exposure, and symptoms of post-traumatic stress disorder (PTSD), depressive symptoms, generalized anxiety symptoms, as well as alcohol and tobacco use. Informed consent was obtained at the initiation of each interview and reaffirmed at the time of biospecimen collection. Participants received $25 compensation upon completion of the telephone interview and were offered an additional $25 incentive for electing to provide a biological specimen. Although DNHS data collection occurred in multiple annual waves (Wave One; 2008–2009, Wave Two; 2009–2010, Wave Three; 2010–2011, Wave Four; 2011–2012, Wave Five 2013; Figure S4) leukocyte-derived miRNA expression data were generated only for selected biospecimen collection waves. For the present analyses, we included DNHS participants (*n* = 185) with complete data on depressive symptom severity and leukocyte-derived miRNA expression at both timepoints, Wave Two (baseline; 2009–2010) and Wave Four (follow-up; 2011–2012). This restriction enabled a two-timepoint longitudinal investigation of whether within-person changes in depressive symptoms were associated with changes in peripheral miRNA expression. This study was conducted in accordance with the Declaration of Helsinki and was approved by the Institutional Review Boards of the University of Michigan and the University of North Carolina.

### 4.2. Assessment of Depressive Symptoms and Demographic Measures

Self-reported demographic information including age, sex, self-reported race, educational attainment, marital status, and employment status was obtained as part of the structured DNHS telephone interview. Depression symptom severity scores were derived from participant responses to the Patient Health Questionnaire-9 [69], which rates each of the nine items on a 4-point scale ranging from 0 (not at all) to 3 (nearly every day), yielding total scores ranging from 0 to 27 [69].

### 4.3. Blood Sampling and RNA Extraction

Protocols for blood sampling and RNA extraction have been described previously [70,71]. Briefly, peripheral whole blood was collected from participants via venipuncture during scheduled in-home phlebotomy visits and processed within two hours. To stabilize leukocyte RNA and prevent degradation, we employed the LeukoLOCK™ Total RNA Isolation System (Thermo Fisher Scientific, Waltham, MA, USA). Whole blood was passed through LeukoLOCK filters, which rapidly capture leukocytes while depleting erythrocytes and platelets. Filters were immediately flushed with RNAlater^®^ solution (Thermo Fisher Scientific, Waltham, MA, USA) to preserve RNA integrity and stored at −20 °C until extraction. This system minimizes ex vivo transcriptional changes and allows for high-quality RNA suitable for transcriptomic profiling. To enable recovery of total RNA, including small RNAs such as mature miRNAs, RNA was isolated using the alternative LeukoLOCK protocol, which employs TRI Reagent^®^ (Thermo Fisher Scientific, Waltham, MA, USA) extraction followed by glass-fiber purification, rather than the standard magnetic bead–based workflow. This alternative protocol is specifically designed to retain RNAs < 200 nt and is recommended by the manufacturer for efficient purification of microRNAs from LeukoLOCK-captured leukocytes. Extracted total RNA underwent stringent quality control. Samples were retained if they met established thresholds of RNA integrity number (RIN ≥ 5.0), 28 S/18 S rRNA ratio ≥ 1.0, and A260/280 absorbance ratio ≥ 1.7 as described previously in [72]. RNA quality metrics, including RIN and 28 S/18 S ratios, were determined using Agilent 2100 Bioanalyzer (Agilent Technologies, Wilmington, DE, USA). The study was approved by the Institutional Review Boards of the University of Michigan and the University of North Carolina.

### 4.4. Small RNA Sequencing and Mapping

A total of 638 RNA samples including 389 from Wave Two and 243 from Wave Four derived from 483 unique participants were selected for small RNA sequencing, as previously described [73]. Library preparation was conducted in accordance with the QIAseq miRNA Library Preparation Kit protocol (QIAGEN, Hilden, Germany), utilizing QIAseq miRNA unique dual indices (Set A–H; QIAGEN). The quality of complementary DNA (cDNA) libraries was assessed using Qubit and TapeStation systems. miRNA sequencing was performed on the Illumina NextSeq 2000 platform (Illumina, San Diego, CA, USA) employing a P3 flow cell with 66 bp single end reads and 10 bp dual indexing. Each sequencing batch included Qiagen XpressRef Universal Total RNA as a positive control. Raw sequencing data underwent quality control using FastQC and MultiQC to assess read quality and adaptor content. Adapter trimming was performed with Trim Galore [74], and paired-end reads were merged using PEAR to reconstruct full-length small RNA sequences. Processed reads were then mapped to the human reference genome (GRCh38) using miRDeep2 [75], which aligns reads and quantifies known and predicted miRNAs. Annotation of precursor and mature miRNAs was based on miRBase (v22) [76]. Only reads aligning to annotated mature strand-specific (-5p and -3p) miRNAs were retained for downstream quantification and expression analysis.

### 4.5. Estimation of Cell-Type Composition and Ancestry Principal Components from Matched DNA Methylation Data

To adjust for cellular heterogeneity, leukocyte proportions, measured at wave 2 and 4, including proportions of CD8+ T cells, CD4+ T cells, natural killer (NK) cells, B cells, monocytes, and neutrophils, were derived from participant and sample-matched EPIC DNA methylation (DNAm) data using IDOL reference implemented in the R/Bioconductor package Epidish [77] and were included as covariates in all differential miRNA analyses. To account for population structure, ancestry principal components were estimated from participant and sample-matched genome-wide DNAm data at wave 2 (i.e., the baseline analytic wave) using the approach described by Barfield et al. [78]. Components were inspected for variance explained and correspondence with self-reported race/ethnicity [78]. The second and third components that best align with ancestry (PC2 and PC3) were retained as covariates in all differential expression models to mitigate confounding by genetic background.

### 4.6. Differential Expression Analysis of miRNA Profiles

Raw read counts for mature strand-specific miRNAs (annotated with “-5p” or “-3p”) were compiled into a count matrix and aligned with corresponding phenotypic data. Only participants with complete paired measurements at both baseline (Wave Two) and follow-up (Wave Four) were retained. Ambiguous or precursor-level identifiers, defined as entries where reads mapped to multiple mature miRNAs, lacked strand-specific “-5p” or “-3p”) annotation, or corresponded to precursor hairpin sequences, were removed to ensure that inference focused on biologically interpretable mature miRNA features.

Counts were imported into edgeR, and low-abundance miRNAs were filtered using filterByExpr with timepoint as the grouping structure to remove features without sufficient information for downstream modeling. Library size differences were then normalized once using the trimmed mean of M-values (TMM) method via calcNormFactors. TMM-adjusted library sizes were subsequently used by voom to compute log_2_-counts-per-million (log_2_-CPM) expression values and observation-level precision weights [79]. Low-abundance miRNAs were filtered using filterByExpr with timepoint as the grouping structure, followed by an additional requirement of CPM > 0.5 in at least 20% of samples, consistent with recommended RNA-seq practice to improve dispersion estimation and reduce false positives [79,80]. This filtering strategy retains features expressed above threshold within each timepoint group, without requiring expression in every matched pair, ensuring that miRNAs with biologically informative patterns are not excluded due to individual-level dropouts.

### 4.7. Model Framework and Specification

Differential expression was evaluated using linear models with empirical Bayes moderation as implemented in limma. We modeled paired miRNA expression measurements (log_2_-CPM) as a function of both within-person symptom change (ΔPHQ-9) and timepoint, while adjusting for baseline depressive symptom severity and covariates. In this repeated-measures framework, ΔPHQ-9 is a person-level predictor that is constant across the two observations for each participant, whereas miRNA expression varies by timepoint. This formulation allows us to test whether individuals who experienced greater symptom change also exhibited corresponding differences in miRNA expression trajectories. For each miRNA, the fitted model was:

Y_ij_ = β_0_ + β_1_·Timepoint_ij_ + β_2_·BaselinePHQ9_i_ + β_3_·ΔPHQ9_i_ + β_4_·Sex_i_ + β_5_·Age_i_ + β_6_·ΔCD8_i_ + β_7_·ΔCD4_i_ + β_8_·ΔNK_i_ + β_9_·ΔBcell_i_ + β_10_·ΔMonocyte_i_ + β_11_·PC2_i_ + β_12_·PC3_i_ + u_i_ + ε_ij_

Here, Y_ij_ denotes the voom-transformed log_2_ (CPM) expression for participant i at timepoint j. The Δ-covariates represent within-person change between baseline and follow-up for immune cell proportions (ΔCD8, ΔCD4, ΔNK, ΔBcell, ΔMonocyte). Ancestry principal components (PC2 and PC3) were included as baseline, static covariates, as genetic ancestry does not change over time.

The term u_i_ captures intra-individual correlation between repeated miRNA expression measurements from the same participant estimated via duplicateCorrelation, and ε_ij_ are residuals weighted by voom to reflect both mean-variance structure and paired design.

Statistical inference focused on the ΔPHQ-9 coefficient (β_3_), which limma reports as logFC. In this framework, logFC (i.e., β_3_) represents the expected change in log_2_ (CPM) miRNA expression per one-point increase in ΔPHQ-9 (i.e., symptom worsening). No manual fold-change transformation between timepoints was applied; “logFC” reflects limma’s internal convention for naming regression coefficients on the log_2_ expression scale [79].

The definition and interpretation of ΔPHQ-9 and ΔPHQ-9 coefficient (β_3_) are as follows:

ΔPHQ-9 = PHQ-9_follow-up_ − PHQ-9_baseline_

Positive values thereby indicate symptom worsening, while negative values indicate symptom improvement.

ΔPHQ-9 coefficient (β_3_) > 0 → higher miRNA expression among individuals whose symptoms worsened.

ΔPHQ-9 coefficient (β_3_) < 0 → higher expression among individuals whose symptoms improved (since ΔPHQ-9 < 0 in improvers).

To this end, ΔPHQ-9 coefficient (β_3_) is interpreted as a regression slope with respect to symptom trajectory, rather than as a classical fold-change between static groups. The directionality of ΔPHQ-9 coefficient (β_3_) therefore reflects the association of each miRNA’s expression with the within-person change in depressive symptoms over time.

Multiple testing was controlled using the Benjamini–Hochberg false discovery rate (FDR) [81]. Results are reported as the ΔPHQ-9 coefficient (β_3_), interpreted as the change in log_2_ (CPM) expression per one-point change in ΔPHQ-9. Positive β_3_ values reflect higher expression with symptom worsening, and negative β_3_ values reflect higher expression with symptom improvement alongside moderated *t*-statistics, *p*-values, and FDR-adjusted *q*-values. Each row in the differential expression output corresponds to a strand-specific mature miRNA feature, typically yielding one coefficient per mature miRNA (Table S1). However, because some mature sequences can be generated by multiple precursor hairpins, the same mature miRNA may appear in more than one row, distinguished by its associated precursor annotation (mature_precursor_ID). AveExpr values reported by limma represent the mean voom-transformed log_2_ (CPM) expression for each miRNA across all included samples (both baseline and follow-up), after filtering and normalization.

### 4.8. Exploratory Target Prediction of Differentially Expressed miRNA

Following differential expression analysis, all nominally associated miRNAs (*p* < 0.05) were subjected to in silico target prediction using the microRNA Data Integration Portal (mirDIP; https://ophid.utoronto.ca/mirDIP/ accessed on 28 February 2026) [82]. mirDIP is an integrative miRNAs–mRNA interaction database that consolidates predictions from more than 30 established microRNA–messenger RNA (mRNA) interaction resources and applies a unified scoring framework that integrates and normalizes confidence values across databases [83]. To improve biological specificity, only interactions in the Very High (top 1%) confidence class were retained for downstream analyses.

Because multiple precursor loci in our dataset could produce the same mature miRNA, mirDIP queries sometimes returned duplicate miRNA–gene pairs. To avoid artificially inflating target counts, predictions were collapsed at the mature miRNA–gene level, retaining the highest available confidence score when minor discrepancies occurred. This deduplication removed only precursor-level redundancy and did not eliminate genuine convergent targeting, in which distinct mature miRNAs regulate the same gene—a recognized feature of miRNA regulatory networks [1,84]. This ensured that cases where several unique miRNAs collectively targeted the same transcript remained fully represented in our dataset. Such convergent regulation was retained because it reflects genuine biological overlaps that may highlight especially important genes under stronger miRNA-mediated control.

To define a consistent reference set for enrichment testing, we also queried mirDIP for the complete set of mature miRNAs that remained after quality control and filtering (*n* = 542), regardless of their association with depressive symptom change. The same stringent Very High confidence threshold was applied, and miRNA–gene pairs were again collapsed at the miRNA–gene level. This background set represents the pool of high-confidence predicted targets possible within our analytic space and provided a stable reference against which enrichment was assessed, controlling for biases in miRNA-target representation within mirDIP.

The final output was used to construct a bipartite mapping of miRNAs to their Very High confidence mRNA targets. Only those miRNAs with at least one retained target gene were carried forward into downstream functional analyses. These filtered miRNA–target pairs were then used as background input for gene-set and pathway enrichment analyses (described below), with the de-duplicated background set from all expressed miRNAs serving as the reference gene list. These analyses are intended to provide functional context for nominally associated miRNAs and should be interpreted as exploratory and hypothesis—generating rather than as evidence of direct miRNA–gene regulatory relationships.

### 4.9. Pathway Enrichment Analyses of Genes Targeted by miRNA

To evaluate whether the observed miRNA expression changes converge on specific biological processes or pathways, we performed Gene Ontology (GO) enrichment analyses in PANTHER (Protein ANalysis THrough Evolutionary Relationships) using genes targeted by miRNAs identified in our study [85]. The PANTHER Statistical Overrepresentation Test (organism: Homo sapiens) was applied using Fisher’s exact test with Benjamini–Hochberg false discovery rate (FDR) correction. Analyses were conducted separately for the Biological Process, Molecular Function, and Cellular Component ontologies, with enrichments considered significant at FDR < 0.05. Additionally, we conducted KEGG pathway [86] enrichment analysis using clusterProfiler on the same foreground set of deduplicated Very High confidence targets, with the custom background set used as reference. Pathways were considered significant at FDR < 0.05.

## 5. Conclusions

This study provides preliminary longitudinal evidence that within-person changes in depressive symptom severity are accompanied by coordinated alterations in peripheral miRNA expression. Although no miRNAs surpassed multiple-testing correction, several candidates including miR-493-3p, miR-409-3p, and miR-323a-3p have previously been implicated in prior preclinical and postmortem studies of depression or stress biology, implicating their potential relevance to stress responsivity, neuroplasticity, and synaptic signaling.

Target prediction and enrichment analyses revealed convergence on molecular networks central to depression biology, such as neurotransmission, neurotrophic signaling and stress–immune regulation, alongside metabolic and circadian modulators. These findings suggest that circulating miRNAs may function as state-sensitive molecular correlates of depressive symptom trajectories, even in a community cohort with relatively low average symptom severity.

Taken together, our results support the utility of genome-scale profiling of miRNAs to potentially uncover dynamic molecular signatures of depressive symptom change. Exploring these alterations can deepen our understanding of the dynamic biology underlying depressive symptom development and may guide the development of future therapeutic targets. Future studies including participants with a broader range of depression scores, greater racial and ethnic diversity, and assessing multiple timepoints that ideally integrate other genomics frameworks, will be critical for validating these candidates in relation to depressive symptom development.

## Figures and Tables

**Table 1:** Demographic characteristics and depressive symptom severity (PHQ-9) of *n* = 185 participants at Waves Two and Four.

Variable	Wave Two	Wave Four
Sex, Female *n* (%)	105 (56.8)	105 (56.8)
Age, mean (SD)	57.23 (14.25)	59.12 (14.25)
Self-reported Race, *n* (%): AA/White/Other	146 (78.9)/29 (15.7)/10 (5.4)	146 (78.9)/29 (15.7)/10 (5.4)
PHQ-9 depressive symptom severity, mean (SD)	4.44 (5.72)	3.96 (5.58)

Values are presented as mean (SD) for continuous variables and *n* (%) for categorical variables. PHQ-9 = Patient Health Questionnaire-9, a measure of depressive symptom severity.

**Table 2:** Top 10 differentially expressed miRNAs (including precursors) associated with changes in depressive symptom severity (ΔPHQ-9).

**-** **Higher Expression in Participants with Symptom Worsening**						
**miRNA (Precursor ID)**	**ΔPHQ-9 coefficient (β_3_)**	**AveExpr**	***t*-Stat**	***p*-Value**	***q*-Value (FDR)**	**Relevance to Depression**
**hsa-miR-363-3p (hsa-mir-363)**	+0.0289	7.42	+3.51	5.06 × 10^−4^	0.0802	Administration of miR-363-3p attenuated post-stroke depressive-like behaviors in stroke rat model [28]
hsa-miR-548at-5p (hsa-mir-548at)	+0.0516	2.77	+3.17	1.40 × 10^−3^	0.0841	No direct evidence in depression (n/a)
**-** **Higher Expression in Participants with Symptom Improvement**						
**miRNA (Precursor ID)**	**ΔPHQ-9 coefficient (β_3_)**	**AveExpr**	***t*-Stat**	***p*-Value**	***q*-Value (FDR)**	**Relevance to Depression**
**hsa-miR-493-3p (hsa-mir-493)**	−0.0564	2.03	−3.49	5.35 × 10^−4^	0.0802	Upregulated in rat hippocampus following stress exposure [29]
**hsa-miR-411-5p (hsa-mir-411)**	−0.0484	3.62	−3.42	6.96 × 10^−4^	0.0802	Upregulated in hippocampal dentate gyrus in rat model following stress exposure, reduced expression following fluoxetine treatment [30]
**hsa-miR-127-3p (hsa-mir-127)**	−0.0501	3.68	−3.38	8.01 × 10^−4^	0.0802	Downregulation associated with Interferon signaling pathway overactivation in kidney disease [31]
**hsa-miR-409-3p (hsa-mir-409)**	−0.0460	4.89	−3.38	8.02 × 10^−4^	0.0802	Downregulated in MDD cases [32]
**hsa-miR-127-5p (hsa-mir-127)**	−0.0604	3.68	−3.38	8.88 × 10^−4^	0.0802	No direct evidence in depression (n/a)
**hsa-miR-432-5p (hsa-mir-432)**	−0.0525	4.23	−3.23	1.25 × 10^−3^	0.0841	Downregulated in MDD cases and in individuals with Anxiety [32]
**hsa-miR-323a-3p (hsa-mir-323a)**	−0.0495	4.01	−3.18	1.39 × 10^−3^	0.0841	Upregulated in MDD cases [33]
**hsa-miR-337-5p (hsa-mir-337)**	−0.0513	3.92	−3.10	2.17 × 10^−3^	0.1083	No direct evidence in depression (n/a)

This table summarizes the 10 miRNAs most strongly associated with within-person changes in depressive symptom severity as measured by ΔPHQ-9. Expression levels were modeled using limma-voom with empirical Bayes moderation, adjusting for baseline PHQ-9, age, sex, DNAm-derived immune cell-type proportions (included as within-person changes, and baseline DNAm-derived ancestry principal components (PC2, PC3), while accounting for repeated measures with duplicateCorrelation. Positive ΔPHQ-9 coefficient (β_3_) values indicate that miRNA expression increased as PHQ-9 scores worsened (higher expression in participants with symptom worsening), whereas negative ΔPHQ-9 coefficient (β_3_) values indicate that miRNA expression increased as PHQ-9 scores improved (higher expression in participants with symptom improvement). AveExpr reflects the mean voom-transformed log_2_ (CPM) expression for each miRNA across all paired baseline and follow-up samples retained after filtering, providing a measure of average abundance. The moderated *t*-statistic represents the estimated coefficient relative to its empirical Bayes-adjusted standard error, with larger absolute values indicating stronger evidence of association. *p*-values are nominal, and *q*-values (FDR) represent multiple testing-adjusted significance estimates. The ‘Relevance to Depression’ column shows studies linking each miRNA to depression, stress models, antidepressant treatment response, or related other related neuropsychiatric or systemic conditions.

## Data Availability

The data presented in this manuscript were generated as part of the first author’s doctoral dissertation, “Leveraging DNA Methylation Profiling and MicroRNA Sequencing to Characterize the Epigenomic Landscape of Major Depressive Disorder Disease Trajectory”. The data supporting the findings of this study have been deposited in the Database of Genotypes and Phenotypes (dbGaP) under accession number phs000560.v2.p1 (dbGaP: https://dbgap.ncbi.nlm.nih.gov/beta/study/phs000560.v2.p1/#study, accessed on 28 February 2026). Access to the controlled data will be granted to qualified investigators for research use consistent with the informed consent of study participants. Requests for access should be submitted through the dbGaP accession page and will be reviewed by the NIH Data Access Committee (DAC) in accordance with NIH Genomic Data Sharing Policy. Investigators may contact the corresponding author for additional information regarding data content and access procedures. Data use is subject to the conditions outlined in the dbGaP Data Use Certification Agreement, including restrictions on re-identification and redistribution.; The R scripts used for our data analysis are available via GitHub (https://github.com/dahrendorff/DNHS-microRNA-expression-and-depressive-symptom-severity-change-.git last access date 28 May 2026).

## Supplementary Materials

The following supporting information can be downloaded at: https://www.mdpi.com/article/10.3390/epigenomes10020035/s1, Figure S1: Top 15 significantly enriched Gene Ontology (GO) biological processes among the predicted target genes of miRNAs whose expression was significantly associated with within-person changes in depressive symptom severity. Figure S2: Top 15 significantly enriched Gene Ontology (GO) molecular functions among the predicted target genes of miRNAs whose expression was significantly associated with within-person changes in depressive symptom severity. Figure S3: Top 15 significantly enriched Gene Ontology (GO) cellular components among the predicted target genes of miRNAs whose expression was significantly associated with within-person changes in depressive symptom severity. Figure S4: Overview of the study design and participant inclusion across waves of the Detroit Neighborhood Health Study (DNHS). Table S1: Differential expression analysis results for all mature miRNAs tested in association with within-person changes in depressive symptom severity. Table S2: Nominally associated miRNAs (*p* < 0.05) identified in the longitudinal differential expression analysis. Table S3: Predicted high-confidence miRNA–target gene interactions for nominally associated miRNAs. Table S4: Gene Ontology (GO) Biological Process enrichment results for predicted target genes. Table S5: Gene Ontology (GO) Molecular Function enrichment results for predicted target genes. Table S6: Gene Ontology (GO) Cellular Component enrichment results for predicted target genes. Table S7: KEGG pathway enrichment results for predicted target genes. Table S8: Enrichment analysis Results of GO cellular components. Table S9. Results of Panther Pathwath analysis. Table S10. Results of KEGG Pathwath analysis.
