# Supplementary material for: Longitudinal Analysis of Peripheral MicroRNA Expression and Depressive Symptom Severity Change in a Community Cohort
Source: Epigenomes. 2026 Jun 2;10(2):35. doi: 10.3390/epigenomes10020035 (PMC13298394; doi:10.3390/epigenomes10020035)
Supplement: Supplementary file 1 [file epigenomes-10-00035-s001.zip › Supplemental_materials.pdf]

Supplementary Materials

Longitudinal analysis of peripheral microRNA expression and depressive symptom severity change in a community cohort.

Authors: Jan Dahrendorff[1], Chengqi Wang[1], Agaz Wani[1], Zachary Graham[1], Allison E. Aiello[2], Annie Qu[3], Derek Wildman[1], Monica Uddin[1]

Affiliations:

1. Department of Global, Environmental, and Genomic Health Sciences, College of Public Health, University of South Florida, Tampa, FL, USA
2. Robert N. Butler Columbia Aging Center, Department of Epidemiology, Mailman School of Public Health, Columbia University, New York, NY, USA
3. Department of Statistics and Applied Probability, University of California, Santa Barbara, Santa Barbara, CA, USA

Keywords:
microRNA expression, depressive symptom severity, genome-scale analysis, community-based cohort

Corresponding author:
Address correspondence to:
Monica Uddin, PhD
University of South Florida
3720 Spectrum Blvd., Suite 304
813-974-9765
monica43@usf.edu

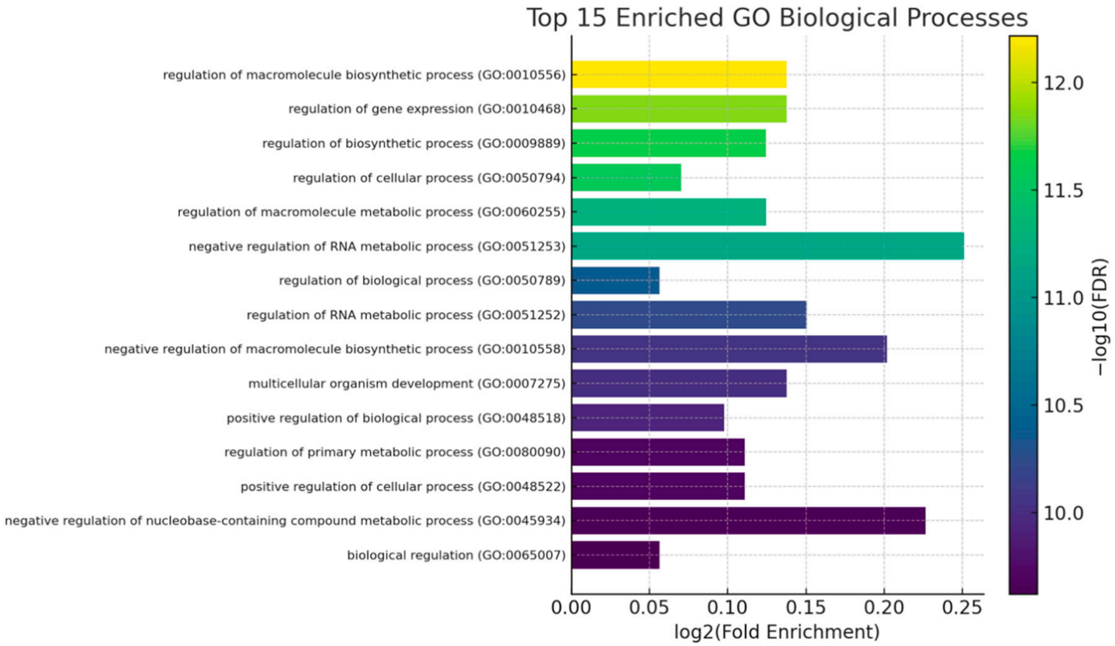

Figure S1 *Top 15 significantly enriched Gene Ontology (GO) biological processes among the predicted target genes of miRNAs whose expression was significantly associated with within-person changes in depressive symptom severity.* Over-representation analyses were conducted using a custom background of all predicted targets of mature miRNAs. Bar length indicates $\log_2$(Fold Enrichment), and bar color represents statistical significance ($-\log_{10}$ FDR).

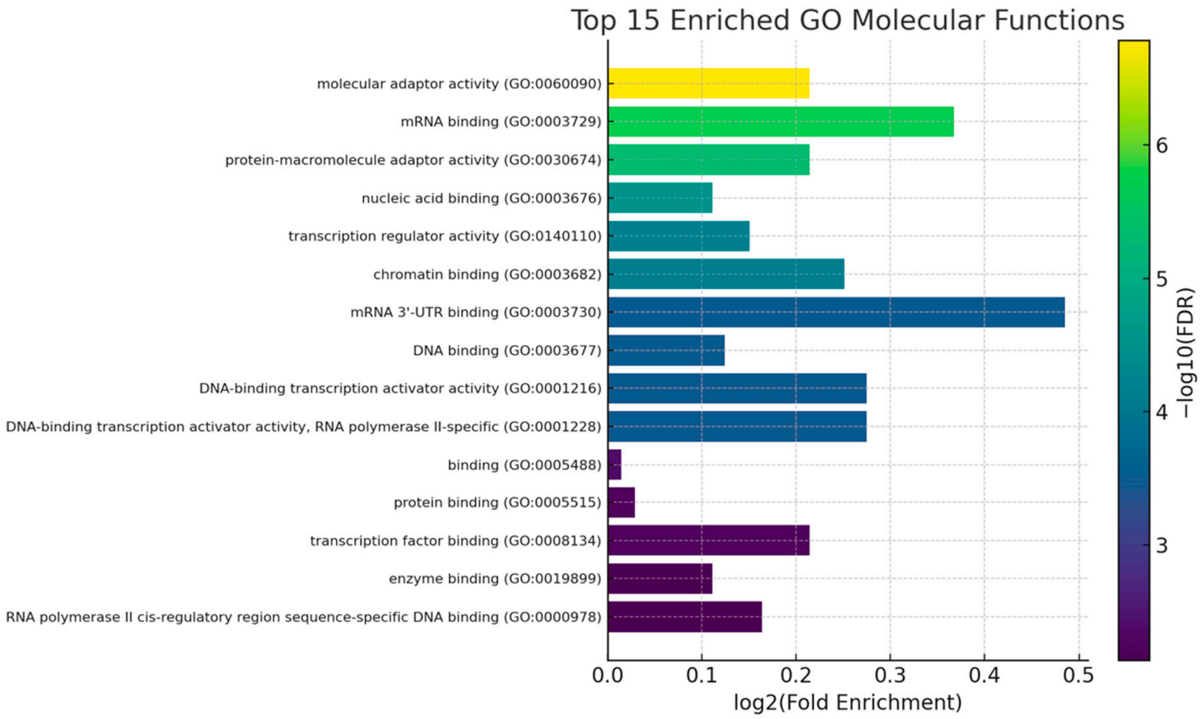

Figure S2 *Top 15 significantly enriched Gene Ontology (GO) Molecular Functions among the predicted target genes of miRNAs whose expression was significantly associated with within-person changes in depressive symptom severity.* Over-representation analyses were conducted using a custom background of all predicted targets of mature miRNAs. Bar length indicates $\log_2$(Fold Enrichment), and bar color represents statistical significance ($-\log_{10}$ FDR).

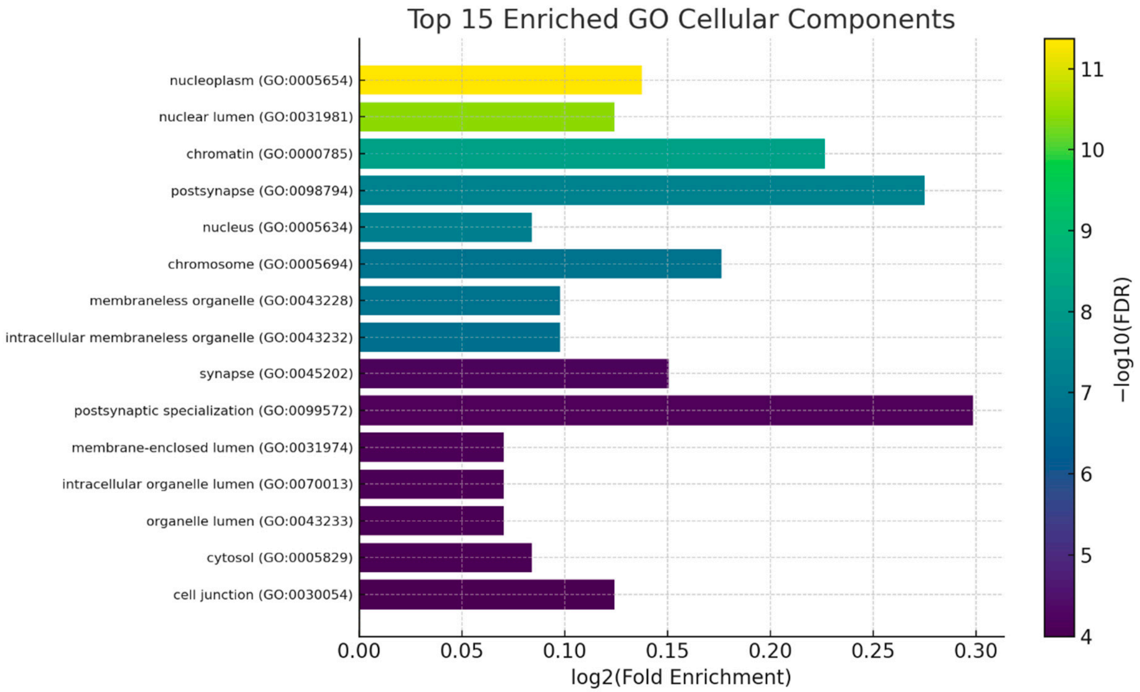

Figure S3 *Top 15 significantly enriched Gene Ontology (GO) Cellular Components among the predicted target genes of miRNAs whose expression was significantly associated with within-person changes in depressive symptom severity.* Over-representation analyses were conducted using a custom background of all predicted targets of mature miRNAs. Bar length indicates $\log_2$(Fold Enrichment), and bar color represents statistical significance ($-\log_{10}$ FDR)

Figure S4 Study Design with Waves

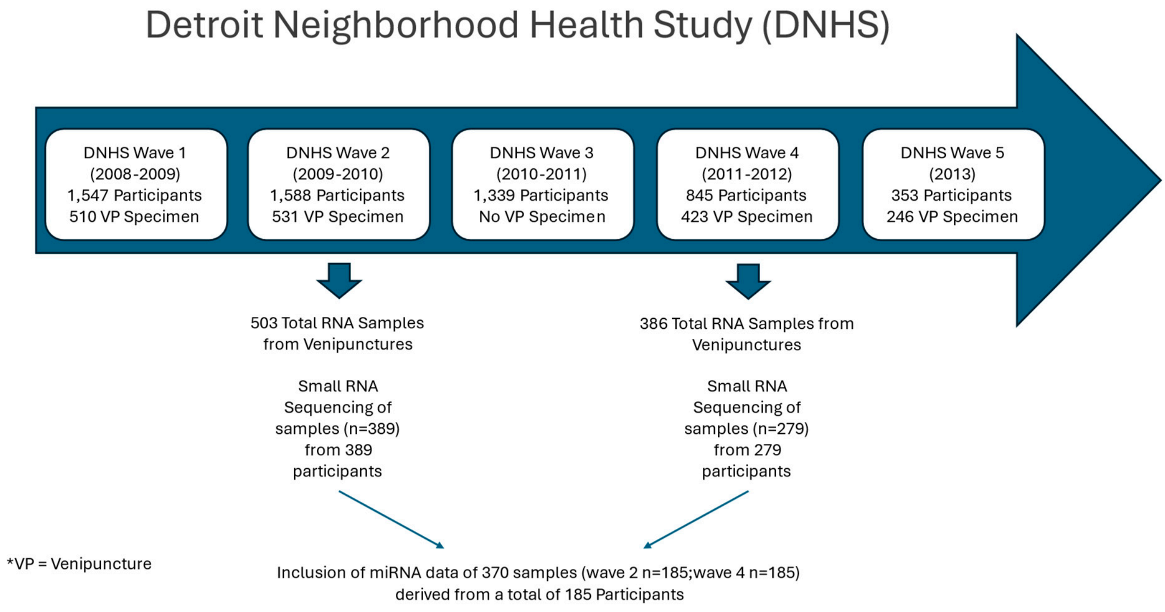

Code Availability

The R code implemented in this manuscript is available on GitHub. dahrendorff/DNHS-microRNA-expression-and-depressive-symptom-severity-change-: Analysis code for Longitudinal analysis of peripheral microRNA expression and depressive symptom severity change in a community cohort.
